# Case of Enteritis

**Published:** 1817-06

**Authors:** Gideon Mantell

**Affiliations:** Member of the Royal College of Surgeons.


*For the London Medical and Physical Journal.*

### *Case of Enteritis;* by Gideon Mantell, Esq. F. L. S. and Member of the Royal College of Surgeons.

J. N. ætat. 53, had for many years been occasionally troubled with dyspepsia. Compelled by his occupation (that of a dealer in hats) to lead a sedentary life, he became very hypochondriacal; and, although at intervals, a walk in the country and cheerful society, rendered him comparatively well and happy, yet these favorable appearances were not of long duration, and were succeeded by derangement of the digestive organs. His appetite was seldom much impaired; and, although his bowels were disposed to costiveness, this was readily prevented by mild aperient medicines. He usually complained of flatulence and a sense of " weight or constriction in the pit of the stomach," unaccompanied by pain or nausea. Infusions of aromatic bitters, with small doses of the sulphate of magnesia, and, occasionally, a few grains of pil. hydrargyri at bed-time, invariably afforded him relief. He had inguinal hernia on the right side, but it was never painful or otherwise troublesome, and he had worn a truss many years without inconvenience.

On the 11th of March he applied for his usual medicines, (infus. cascarillæ cū magnes. sulph.) and, to use his own expression, " was well in a few days."

On April the 13th, he felt himself particularly well, and retired to rest at ten o'clock in the evening. He immediately fell into a sound sleep, and reposed till four in the morning of the 14th, when he was awakened by acute pain, accompanied with distension in the umbilical region extending to the right hypochondrium: he drank some warm rum and water; but, the pain increasing, he sent for me at half past five. I found him lying across the bed, with a countenance expressive of great anxiety. On seeing me, he exclaimed, " the wind has stopped in my stomach—I must die—nothing will do me any good!" His pulse was ninety, weak and faultering; the temperature of the skin but little augmented; the tongue clean; respiration hurried; and the abdomen so remarkably tense, that it resisted the firmest pressure pressure I could make, and which did not appear to aggravate his sufferings. Aware of the existence of hernia, my first inquiries were directed with a view to ascertain how far it might be connected with the present symptoms: he told me, that he had passed a copious alvine discharge the preceding evening, and had removed the truss when he went to bed, that the rupture was now full of wind, but quite easy. On examination, I found the scrotum considerably distended by the hernia; it was elastic, and could be handled without producing any uneasiness. I carefully attempted its reduction, but could not succeed. Fomentations to the abdomen, and emollient laxative clysters, were directed, with an aperient mixture, (magnes. sulph. ℥j. aq. f. ℥iij. m. capiat. cochl. iv. omni hora donec alvus respondeat.) At eight, he was no better; the pain in the abdomen had increased; the medicines had not operated; and the hernia was rather painful. His pulse was full, and one hundred and ten per minute; ℥xviij. of blood were taken from the arm, it flowed very rapidly, and occasioned a slight fainting; I again had recourse to the taxis, but was unsuccessful. The warm-bath was immediately directed: after he had been in it twenty minutes, the pulse, which had been diminished in frequency and strength by the previous bleeding, became full, and rose to one hundred and twenty; venæsection was repeated, but, scarcely had three ounces of blood escaped, when he fainted. I seized this favorable opportunity, and, to my great joy, reduced the hernia in an instant. It may be proper, in this place, to remark, that the blood exhibited no marks of inflammation; not the least trace of a buffy hue or cup-like appearance was perceptible. The reduction of the hernia afforded no relief; his pulse was scarcely perceptible; the pain and constipation continued. Three doses of cathartic pills, consisting of ext. colocynth. c. calomel and ext. hyosciami were ordered to be taken at intervals of half an hour between each dose, and a large blister applied to the abdomen.

The means made use of were attended with no advantage, nothing appeared to arrest the fatal progress of the disease; I, therefore, forbear to occupy the pages of your excellent Journal, by an unnecessary detail of the treatment. At five o'clock, his pulse was one hundred and twenty, soft and distinct. I had left him but a very few minutes, when his servant came to require my immediate attendance—before I arrived, he was no more!

I could not obtain permission to examine the body till the 17th. On opening the cavity of the abdomen, (in the presence sence of Messrs. Moore and Hodson, who had attended with me,) a considerable quantity of fluid gushed out, and we discovered adhesions so firm and extensive, between the omentum and peritoneum, that it was impossible to separate them from each other without laceration. Marks of inflammation were general on the peritoneal coat of the abdominal viscera. The stomach, the small and large intestines, particularly the cœcum, the omentum, peritoneum, and external surface of the liver, exhibited large patches of a florid red colour, and a layer of coagulable lymph covered many parts of the peritoneum; the inferior margin of the omentum adhered to the hernial sac, and it seems probable that this circumstance might have occasioned the uneasiness and constriction of the stomach, of which the patient had so long complained. There was no appearance that strangulation had existed; the portion of intestine which formed the hernia, had fewer traces of inflammatory action than any other part of the intestinal canal. An inspection of the stomach enabled us to discover the origin of the fluid observed on dividing the parietes of the abdomen; it had escaped from the stomach through a small ulceration near the pylorus; the margin of this ulcer was of a very dark colour, and the villous coat of the stomach surrounding it, possessed considerable redness. The kidneys were healthy; the pancreas and spleen were not examined. In the thorax, some marks of inflammation were also observed; the pleura pulmonalis and costalis adhered to each other almost throughout their whole extent. The heart was free from disease.

In offering the preceding case to the notice of your readers, I have confined myself to a plain statement of facts. I beg permission, however, to observe, that the following appear to be the most striking features of the case:—

1. The ulceration of the stomach, with the absence of vomiting and nausea.

2. The extent of inflammatory action; the adhesions which it produced; the rapidity of its progress; and its fatal termination, without occasioning gangrene.

3. The healthy appearance of the blood taken in a full stream, and from a large orifice, when active inflammation was present.

*Castle-place, Lewes;*

*May* 5, 1817.

